# Differences in the Expression of Autophagy Markers Microtubule-Associated Protein Light Chains 3A and 3B in Oral Premalignant Lesions and Oral Squamous Cell Carcinoma: A Cross-Sectional Study

**DOI:** 10.7759/cureus.68994

**Published:** 2024-09-09

**Authors:** Tulika Wakhloo, Prashant Durgapal, Nilotpal Chowdhury, Srinivas Reddy, Ashi Chug, Sanjeev Kishore

**Affiliations:** 1 Dentistry, All India Institute of Medical Sciences, Rishikesh, Rishikesh, IND; 2 Pathology and Laboratory Medicine, All India Institute of Medical Sciences, Rishikesh, Rishikesh, IND; 3 Oral and Maxillofacial Surgery, GSR Institute of Craniomaxillofacial and Facial Plastic Surgery, Hyderabad, IND

**Keywords:** autophagy, immunohistochemistry, microtubule-associated protein, oral premalignant lesions, oral squamous cell carcinoma

## Abstract

Background

Microtubule-associated protein light chains (LC) 3A and 3B are the structural proteins of the autophagosomal membrane widely used as endogenous autophagy markers. LC3A and LC3B autophagosomes reportedly have a distinct subcellular localization yet their role in the transition from premalignant to malignant phase remains unclear. This exploratory study aimed to investigate the expression of autophagy-related proteins LC3A and LC3B in oral premalignant lesions (OPL) and oral squamous cell carcinoma (OSCC) cases.

Methodology

A cross-sectional study was conducted on 100 OPL and 39 OSCC samples. OPL samples comprised both dysplastic and non-dysplastic lesions. The expression of LC3A and LC3B markers was evaluated in the study samples using immunohistochemistry and associated with dysplasia in OPL and with invasive OSCC versus OPL. Fisher’s exact test was used for statistical analysis.

Results

There was a higher ratio of LC3A positivity in non-dysplastic OPL (31/38) compared to dysplastic premalignant lesions (36/62, p=0.017). There was a higher ratio of LC3B positivity in dysplastic OPL (16/62) compared to non-dysplastic lesions (4/38) with a trend towards statistical significance (p=0.075). There was no statistical difference in the ratio of LC3A positivity between OSCC (23/39) and premalignant (67/100) lesions, while the ratio of LC3B marker positivity was higher in OSCC cases (18/39) relative to premalignant lesions (20/100, p=0.003).

Conclusion

Autophagy-related proteins LC3A and LC3B may have different roles to play in a disease context manner. LC3A is likely to be negatively associated with dysplasia in OPL while LC3B expression is positively associated with carcinogenesis of OSCC, possibly including dysplasia.

## Introduction

Autophagy is a highly conserved lysosome-mediated programmed cell survival mechanism vital to cellular homeostasis under both physiologic and pathologic conditions. This major intracellular catabolic pathway reportedly involves the removal of damaged intracellular proteins and organelles by the autophagosome. The autophagosome fuses with the lysosome and the engulfed contents are degraded by the lysosomal enzymes producing macromolecules which are recycled and reused by cells [1-4]. Oral squamous cell carcinoma (OSCC) is a fatal malignancy and India accounts for about one-third of the total global burden [5,6]. It may be preceded by visible changes in the oral mucosa known as the oral premalignant lesions (OPL) which can regress, remain unchanged or undergo malignant transformation [7]. The most commonly described OPL are leukoplakia, erythroplakia, lichen planus, and oral submucous fibrosis [8]. These lesions can be non-dysplastic or dysplastic with the risk of neoplastic transformation being higher in dysplastic lesions [7,8]. Deregulation of autophagy has been linked to a broad spectrum of human diseases, however, its role in oral carcinogenesis is less understood. Understanding the advances in oral cancer biology is imperative to develop effective strategies and counteract its reportedly increased incidence in young adults [9-12].

The structural proteins of autophagosomal membranes, widely used as endogenous autophagy markers, are microtubule-associated protein light chain (LC) 3A, 3B and 3C. The expression of LC3C is reported to be much lower than that of LC3A and LC3B in all tissues [4]. Most previous studies have associated the overall increase in LC3 expression with OSCC; however, whether autophagy is associated with the transition from premalignant to malignant phase remains unclear. This question is further complicated by the discovery that LC3A and LC3B autophagosomes have a distinct subcellular localisation and are never composed by both proteins suggesting that both LC3 isoforms, LC3A and LC3B may act differently [1]. Therefore, the present exploratory cross-sectional study aimed to investigate the association between LC3A and LC3B immunopositivity with dysplasia in OPL and the association between LC3A and LC3B immunopositivity with OSCC versus OPL using immunohistochemistry (IHC).

## Materials and methods

**Figure 1:**
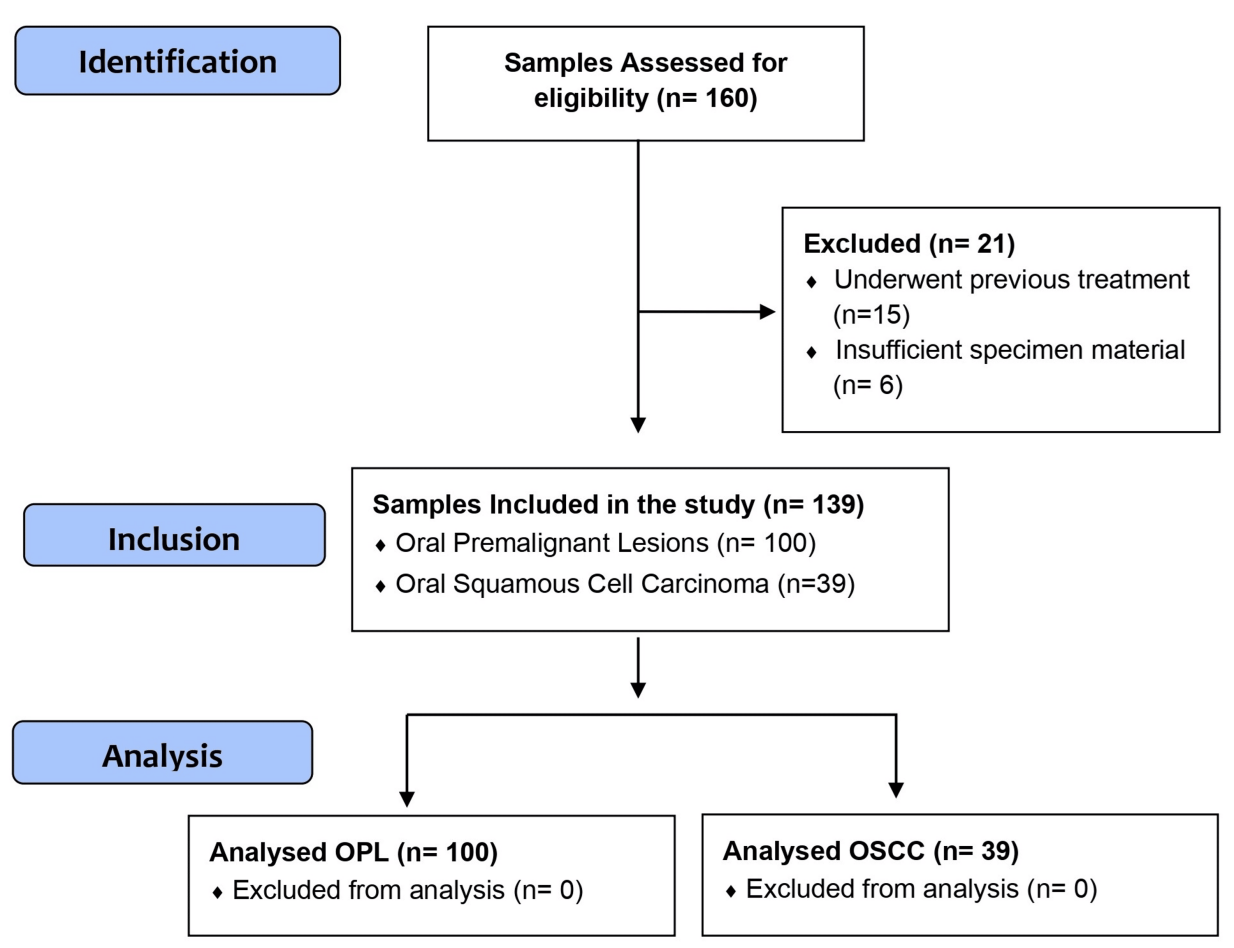
STROBE flow diagram STROBE: STrengthening the Reporting of OBservational studies in Epidemiology; OPL: oral premalignant lesion; OSCC: oral squamous cell carcinoma

This cross-sectional observational study was performed on 100 OPL samples (38 non-dysplastic and 62 dysplastic lesions), and 39 OSCC samples obtained by convenience sampling from archived specimens of various intra-oral sites from the Departments of Dentistry, Otorhinolaryngology, and Surgical oncology between 2018 and 2022. The studied specimens were of patients who did not undergo any kind of treatment and were histopathologically proven cases of OPL and OSCC with sufficient specimen material from which multiple paraffin sections could be made. All OPL and OSCC specimens were diagnosed and classified according to World Health Organisation criteria by a pathologist. Clinical data was obtained from histopathology requisition forms, histopathology reports, and medical records. The study was approved by the Institutional Review Board and Institutional Ethics Committee (AIIMS/IEC/19/724). The manuscript was prepared following uniform reporting guidelines for observational studies (Figure 1).

IHC procedure

Tissue sections, 4 μm thick, obtained by sectioning formalin fixed paraffin embedded blocks were deparaffinized in xylene and rehydrated in descending grades of ethanol. Antigen retrieval was done by soaking the sections in Tris Ethylenediaminetetraacetic (EDTA) acid buffer (pH=8.5 to 9) and placing them under steam pressure for 15 minutes using PathnSitu (multiple epitope retrieval system) (Thermo Fisher Scientific, Waltham, USA). Endogenous peroxidase was blocked with 3% hydrogen peroxide at room temperature. After washing with Tris-buffered saline auto wash solution (pH=7.4 to 7.6), the sections were incubated with 1:100 dilution of primary antibodies LC3A and LC3B (Invitrogen, Thermo Fisher Scientific) with catalogue number: PA5-22990 and PA5-85081 respectively. The secondary antibody (Medaysis, Livermore, USA) was then used. Finally, the reaction products were visualised using 3,3′ Diaminobenzidine (Medaysis) for five minutes, followed by hematoxylin counterstaining and mounting with Dibutylphthalate Polystyrene Xylene (Central Drug House Pvt. Ltd., New Delhi, India). The human brain was used as a positive control for both LC3A and LC3B. Negative controls were obtained by omitting the addition of primary antibodies.

Evaluation of IHC

The slides were scored in a blinded fashion independently by two investigators under a light microscope. The immunoexpression of LC3A and LC3B was evaluated for the proportion of positive cells and intensity of staining at high magnification (20x and 40x). The proportion of specimens showing positive staining was graded from 0 to 3 according to the following criteria (cellularity score): 0 - absent, 1 - less than 10% tumour cells stained, 2 - 10% to 50% tumour cells stained and 3 - more than 50% tumour cells stained. The intensity was graded as 0, 1+, 2+, and 3+ for negative, weak, moderate, and strong staining respectively (intensity score). The total score ranged from 0 to 9 and was obtained by multiplying the cellularity score with the intensity score [13,14].

Statistical analysis

The data were statistically analysed using the R software version 4.0.3 (The R Foundation For Statistical Computing, Vienna, AT). The association of LC3A and LC3B immunoexpression with OPL versus OSCC was estimated using Fisher’s exact test. In the subset of pre-malignant lesions, the association of LC3A and LC3B with dysplasia was estimated using Fisher’s exact test. The odds ratios were calculated with 95% confidence intervals and a p-value less than 0.05 was considered statistically significant. A convenient sample size of 100 OPL and 39 OSCC cases was taken.

## Results

A total of 100 OPL and 39 OSCC small biopsy specimens were included in this study. The OSCC cases comprised 10, 27 and two cases of well-differentiated, moderately differentiated and poorly differentiated OSCC, respectively. The distribution of all cases of OPL with demarcation to dysplastic and non-dysplastic cases is given in Table 1. The clinical information of all cases is summarised in Tables 1-2. The comparative evaluation of LC3A and LC3B expression in OPL versus OSCC samples is also given in Table 2 while the comparison of LC3A and LC3B in non-dysplastic versus dysplastic OPL cases is summarised in Table 3.

**Table 1:** Frequencies of histological diagnosis of oral premalignant lesion cases

Diagnosis	Number (n=100)	% of Total
Non-dysplastic Lesions		
Oral Keratosis	7	7
Hyperplasia	19	19
Lichenoid Tissue Reaction	4	4
Lichen Planus	3	3
Verrucous Hyperplasia	4	4
Oral Submucous Fibrosis	1	1
Dysplastic Lesions		
Mild Dysplasia	21	21
Moderate Dysplasia	2	2
Severe Dysplasia	30	30
Leukoplakia	9	9

**Table 2:** Comparative evaluation of clinical features, LC3A and LC3B marker expression in oral premalignant lesions and oral squamous cell carcinoma samples OPL: oral premalignant lesion; OSCC: oral squamous cell carcinoma; LC: light chain; OR: odds ratio; C.I.: confidence interval

Variables	Outcomes	OPL (n=100)	OSCC (n=39)	p-value	OR [95% C.I.]
Age; n (%)	≤40 years	28 (28)	13 (33)	0.5	-
	>40 years	72 (72)	26 (67)		
Gender; n (%)	Male	93 (93)	39 (100)	0.2	-
	Female	7 (7)	0 (0)		
LC3A (2 tier); n (%)	Negative	33 (33)	16 (41)	0.4	0.71 [0.31, 1.64]
	Positive	67 (67)	23 (59)		
LC3B (2 tier); n (%)	Negative	80 (80)	21 (54)	0.003	3.39 [1.42, 8.18]
	Positive	20 (20)	18 (46)		

**Table 3:** Comparative evaluation of LC3A, LC3B marker expression in non-dysplastic and dysplastic oral premalignant lesions LC: light chain; OR: odds ratio; C.I.: confidence interval

Marker	Outcome	Non-dysplasia (n=38)	Dysplasia (n=62)	p-value	OR [95% C.I.]
LC3A; n (%)	Negative	7 (18)	26 (42)	0.017	0.32 [0.10, 0.88]
	Positive	31 (82)	36 (58)		
LC3B; n (%)	Negative	34 (89)	46 (74)	0.075	2.93 [0.84, 13.12]
	Positive	4 (11)	16 (26)		

**Figure 2:**
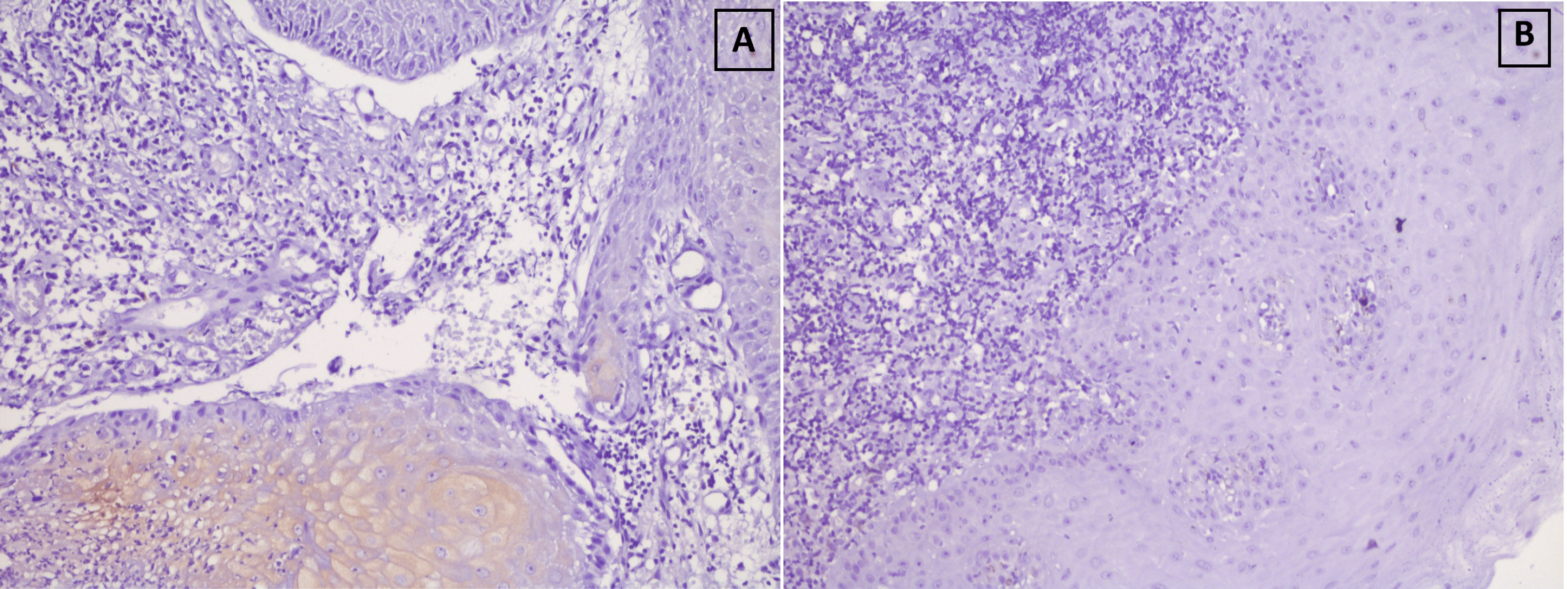
Immunohistochemical stains in representative non-dysplastic OPL sections (20x). (A) LC3A positivity. (B) LC3B negativity (with positive internal control) OPL: oral premalignant lesion; LC: light chain

**Figure 3:**
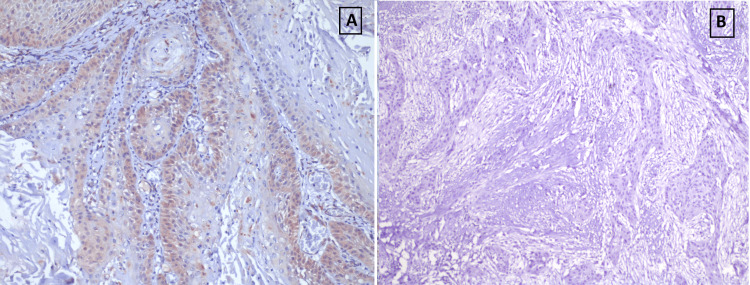
Immunohistochemical stains in representative OSCC sections. (A) LC3B positivity 20x. (B) LC3A negativity (10x; with positive internal control) OSCC: oral squamous cell carcinoma; LC: light chain

**Figure 4:**
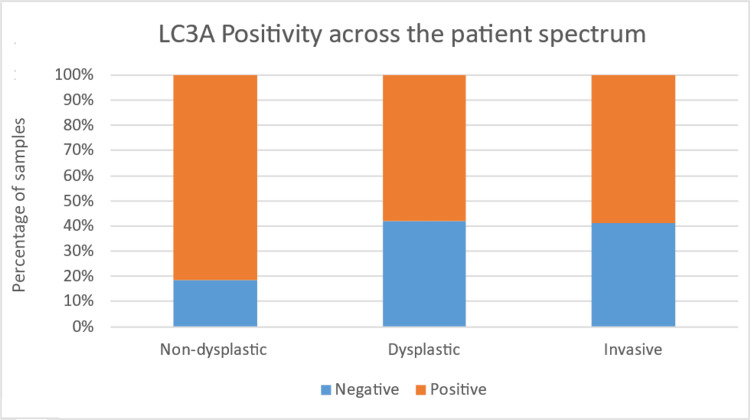
LC3A positivity across the patient spectrum LC: light chain

**Figure 5:**
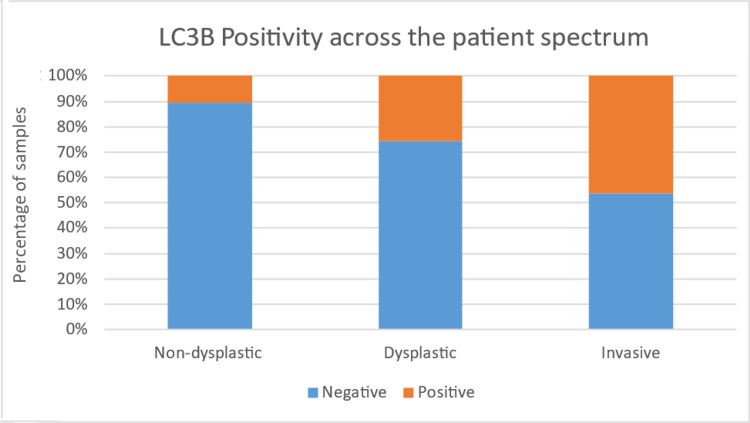
LC3B positivity across the patient spectrum LC: light chain

The present study found that LC3A positivity was negatively associated with dysplasia and LC3B positivity was positively associated with dysplasia in OPL cases, with a trend towards statistical significance. The LC3A expression in non-dysplastic OPL was observed in the superficial layers rather than the basal layer of the epithelium and was punctate cytoplasmic in pattern (Figures 2A, 2B). For OSCC cases, LC3B positivity, but not LC3A positivity was found to be positively associated and was observed in the basal layer compared to superficial layers of the epithelium and was punctate cytoplasmic in pattern (Figures 3A, 3B). The graphical representation of the same has been illustrated in Figures 4, 5.

## Discussion

OPLs are relatively common, occurring roughly between 1.5% and 4.5% of the world’s population and are important targets in cancer prevention [15]. Since these lesions account for 17% to 35% of all new OSCC cases, early detection especially in high-risk groups is important to prevent further morbidity [15,16]. The present study found increased LC3A expression in non-dysplastic OPL compared to dysplastic pre-malignant lesions. The opposite was true for LC3B. The increased LC3A expression in non-dysplastic OPL was observed in the superficial layers of the epithelium rather than the basal layer. This suggests that LC3A and LC3B have different roles, with LC3B possibly being associated with carcinogenesis compared to LC3A. To the best of our knowledge, no previous studies have compared both LC3A and LC3B in OPL to draw comparisons. The expression of LC3A in premalignant lesions has also not been evaluated to the best of our knowledge.

One study did report increased LC3B expression in 47 verrucous hyperplasia relative to normal oral mucosa which is in contrast to our study results [4]. In the literature review, two studies reported increased LC3-II markers in oral leukoplakia and oral submucosal fibroid lesions respectively when compared with controls [7,17]. It is important to emphasize that during the process of autophagy, both LC3A and LC3B can generate the lipidized LC3-II form (LC3A-II and LC3B-II) which is the main component of the autophagosome membrane [1,4]. Therefore, the use of non-specific antibodies can show an increased LC3-II expression due to dual recognition of LC3A and LC3B isoforms.

The present study found that LC3B was associated positively with OSCC while LC3A was not. The increased LC3B positivity in invasive OSCC was observed in the basal layer compared to the superficial layers of the epithelium. This further supports our contention that LC3A and LC3B may have opposing roles to play; both a pro as well as anti-carcinogenic role, despite being isoforms of the same protein suggesting a very important regulatory role for this protein in oral squamous lesions. However, since the individual biological roles of LC3A, B and C sister proteins reportedly remain obscure, cell culture studies are required to confirm our findings. In addition, quantitative methods like RT-PCR can be further used to assess the expression of LC3A and LC3B markers on a genomic level. The previous studies in OSCC cases have investigated and found increased LC3, LC3B, and LC3-II marker expression in OSCC respectively [4,9,18,19]. However, earlier studies did not compare different isoforms. The clinical implication of the present study is that, rather than LC3A or non-specific LC3, LC3B may be used as a biomarker for dysplastic OPL and OSCC.

Limitations of the study

Increased LC3A or B expression not only indicates increased autophagosome formation and activated autophagy but it could also reflect relative suppression of subsequent steps, that is, the inability of autophagosomes to fuse with lysosomes. Monitoring the simultaneous autophagic flux using the p62 (Sequestosome-1) marker in a subsequent study is needed to overcome this limitation. Furthermore, in our study, normal mucosa was not included, due to ethical concerns of obtaining such samples.

## Conclusions

The present study concluded that LC3A and LC3B play different roles in a disease context manner. LC3A is likely to be associated with OPL and LC3B expression with OSCC. The findings of this study emphasize the need for conducting further studies to evaluate the role of LC3A and LC3B in early diagnosis and developing treatment modalities for OPL and OSCC.
